# Appendicitis Caused by Endometriosis Within the Bowel Wall

**DOI:** 10.7759/cureus.9614

**Published:** 2020-08-08

**Authors:** Anupam K Gupta, Adam Mann, Avraham Belizon

**Affiliations:** 1 Minimally Invasive Surgery, University of Miami Hospital, Miami, USA; 2 General and Colorectal Surgery, Boca Raton Regional Hospital/Florida Atlantic University, Boca Raton, USA

**Keywords:** appendicitis, endometriosis, endometrioma, appendiceal endometriosis

## Abstract

Acute appendicitis is one of the most common causes of acute abdominal pain seen in the emergency room. Common etiologies include obstructing appendicolith and lymphoid adenopathy. Appendiceal endometriosis is rare and typically involves the serosal layer. This case report describes an unusual case of appendicitis secondary to endometriosis in the musclularis mucosa of the appendix in the 36-year-old lady with no prior history of endometriosis.

## Introduction

Acute appendicitis is the most common surgical diagnosis of abdominal pain in the right lower quadrant [1]. Endometriosis in the right lower quadrant can mimic acute appendicitis and can deposit into the nearby tissue such as the ovary, fallopian tube, and the appendix [2,3]. Endometrial tissue is in the appendix in 0.4% of the general population and 2.8% of patients with previously diagnosed endometriosis [4]. Appendiceal endometriosis most commonly deposits onto the serosal layer of the intestinal wall with occasional involvement of deeper layers [5]. Endometrial tissue in the muscularis propria without any involvement of the serosa layer/surrounding structures causing appendicitis is a histopathological diagnosis we encountered in this case.

## Case presentation

**Figure 1:**
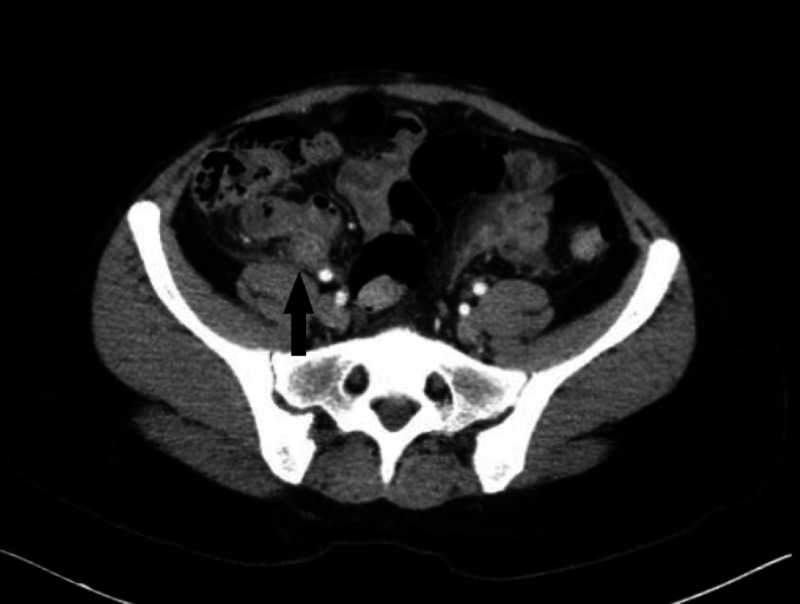
Retrocecal inflamed appendix adhered to the cecum

**Figure 2:**
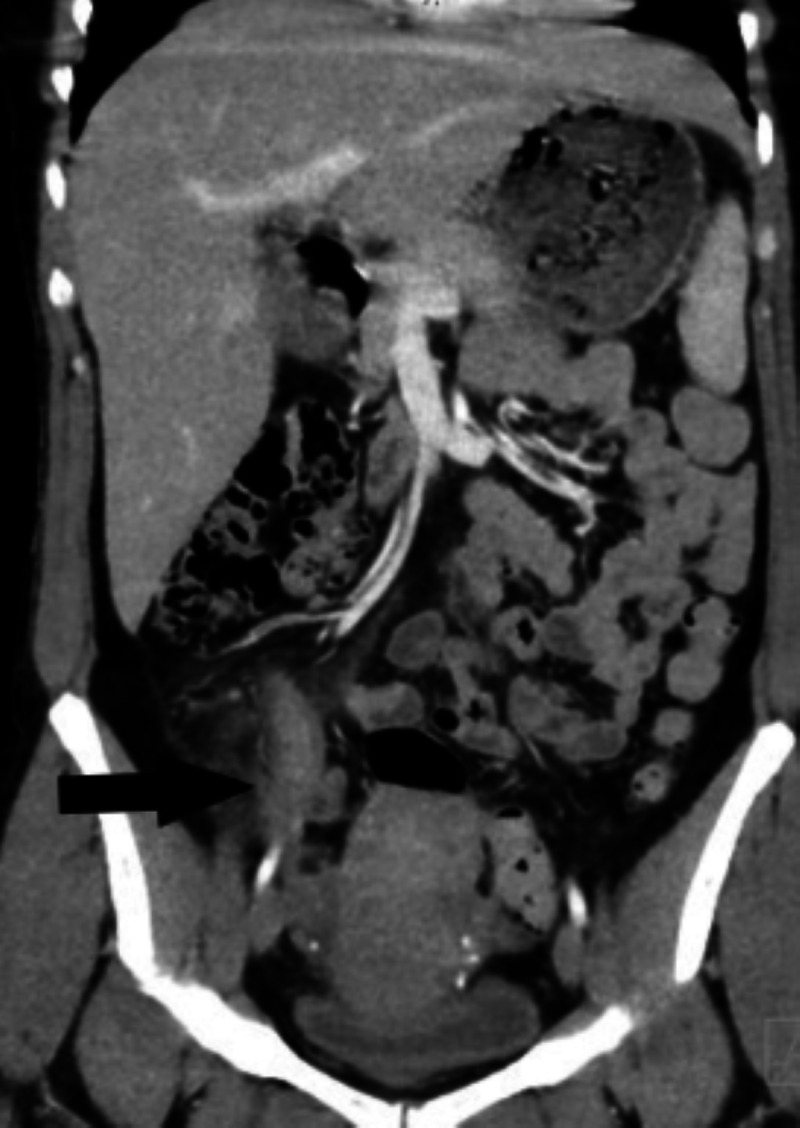
1.6 cm appendix with periappendiceal inflammatory changes

A 36-year-old woman with no prior medical history presented to the emergency room with a one-day onset of acute abdominal pain. She had pain and tenderness in the right lower quadrant. On arrival, her vital signs were stable, and clinical examination was positive for pain and tenderness in the right lower quadrant. Blood work showed evidence of leukocytosis of 11.7 k/ml. Computed tomography of the abdomen and pelvis performed in the emergency room showed evidence of a dilated 1.6 cm retrocecal appendix with periappendiceal inflammatory changes (Figures 1, 2).

 

**Figure 3:**
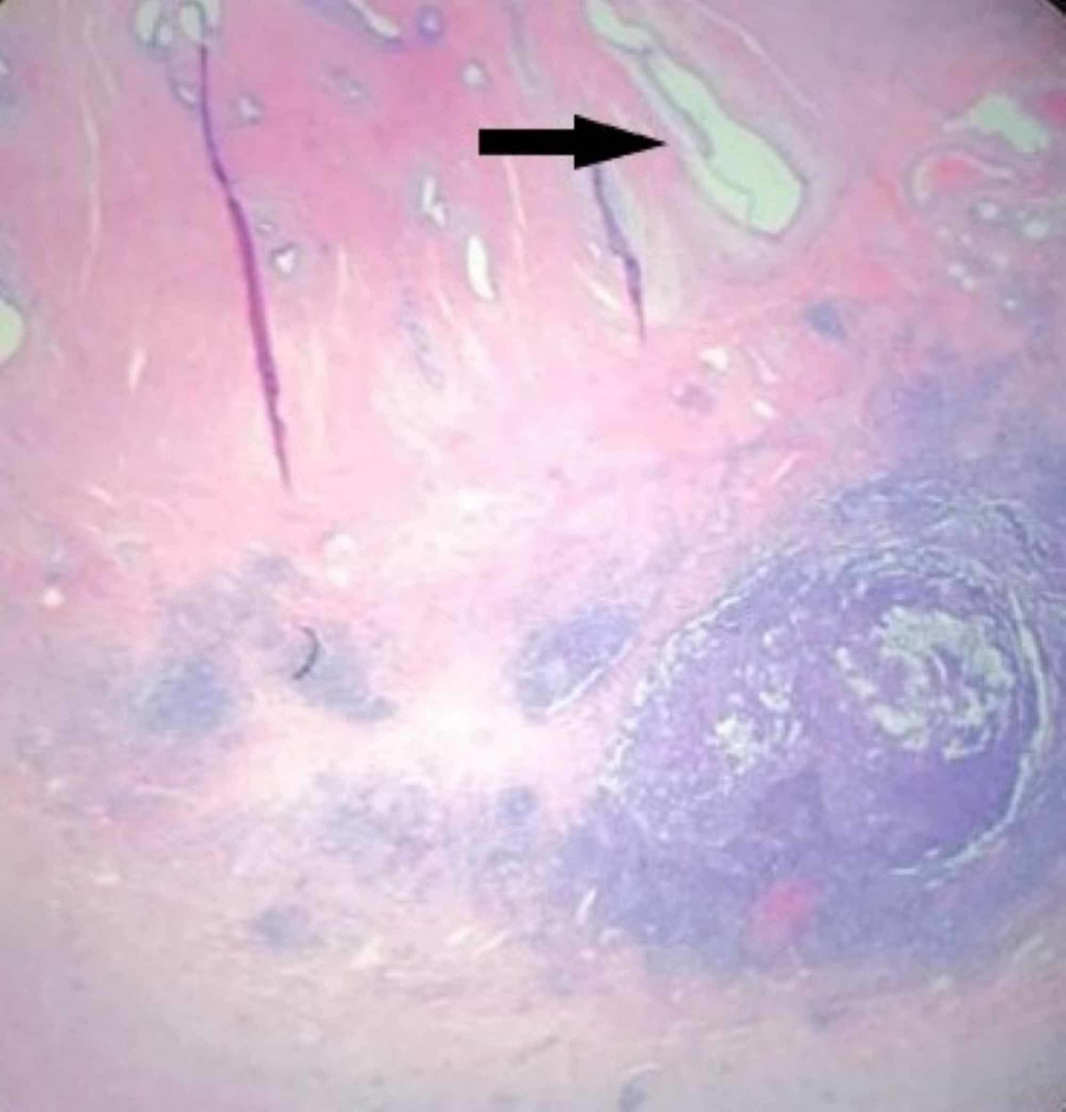
Appendix, arrow depicts the endometrial glands and stroma and inflamed lymphoid tissue inferiorly

**Figure 4:**
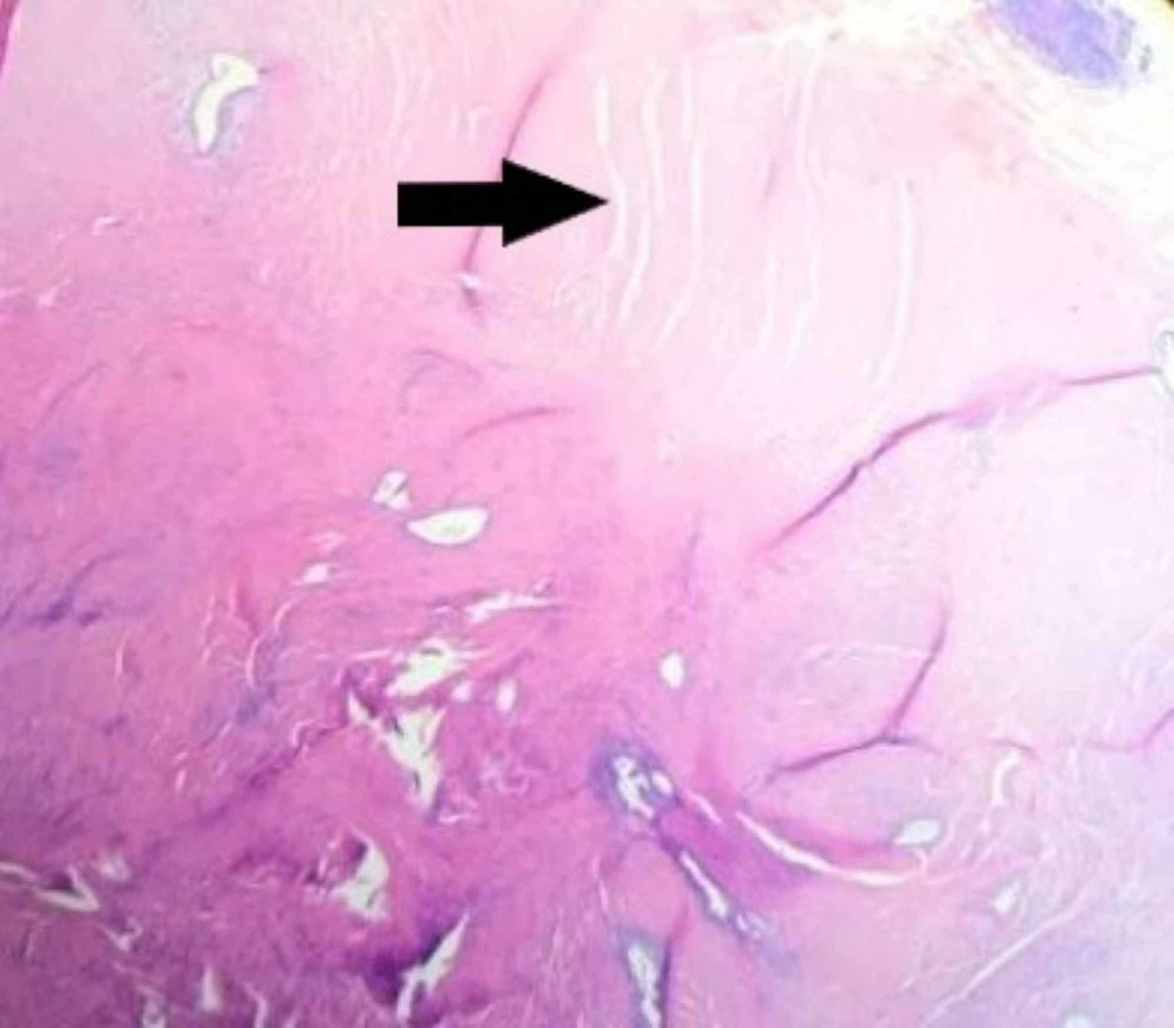
Cecal wall. Arrow points to an enlarged muscularis propria with endometrial glands and stroma

**Figure 5:**
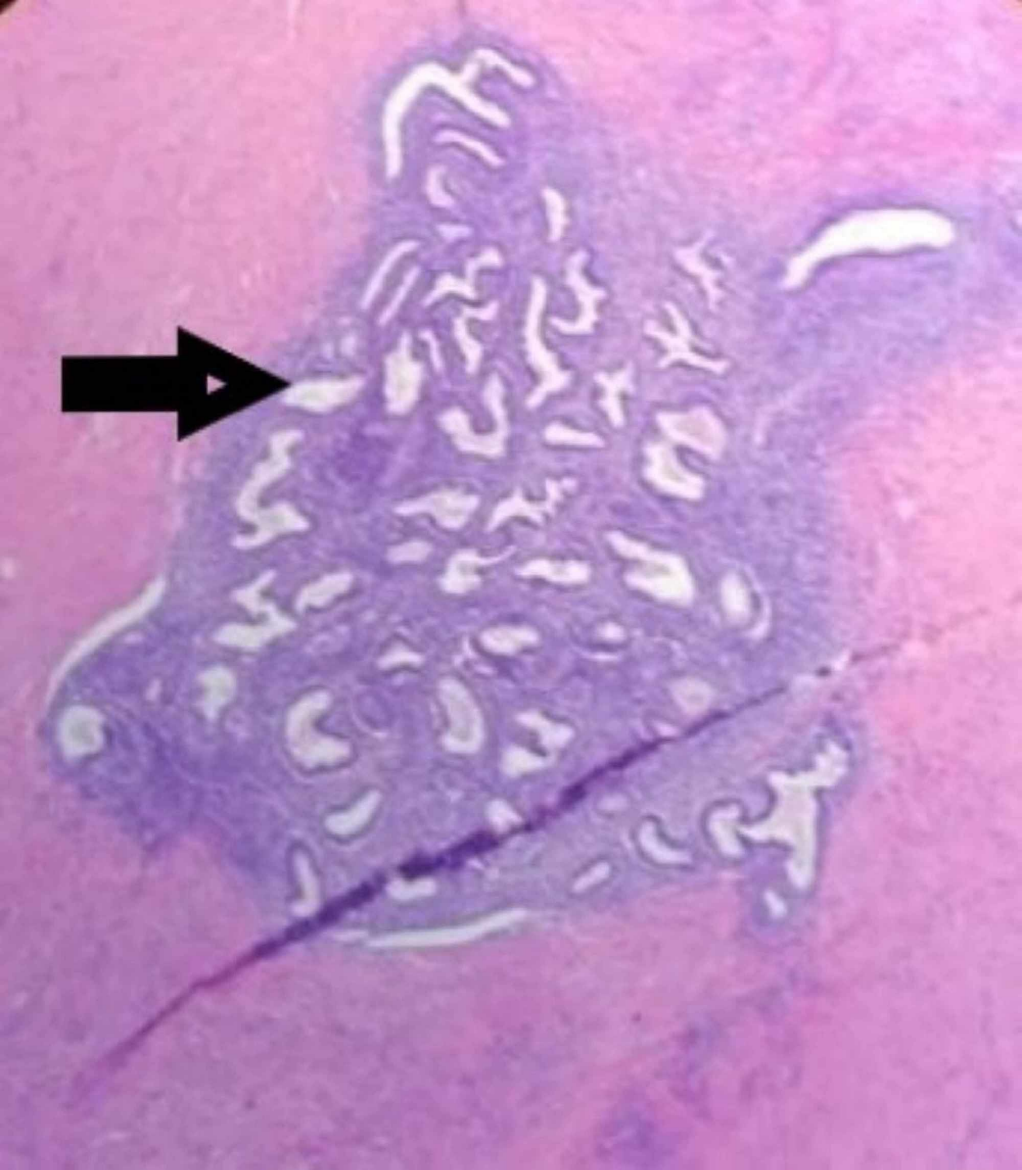
Endometrial glands in the muscularis mucosa of the cecum

The patient underwent an emergent laparoscopic appendectomy. In the operating room, the cecum appeared to be edematous and inflamed, and we performed an open ileocecectomy with primary ileocolic anastomosis. The postoperative course was uneventful. On postoperative day two, the patient tolerated a clear liquid diet and on day five postoperative she was discharged from the hospital. Histopathology analysis of the specimen revealed the presence of endometriosis of the muscularis propria of the cecum and the appendix with findings consistent with appendicitis (Figures 3, 4, 5).

## Discussion

Endometriosis is a benign disease in females characterized by an abnormal growth of endometrial glands and stroma outside of the uterine cavity. Women of childbearing age can have endometriosis, and this ectopic tissue undergoes normal physiologic response to female reproductive hormones, which can irritate surrounding tissues [6]. The most common sites of endometriosis are the ovaries, fallopian tubes, pelvic peritoneum, cervix, vagina [2,7,3].

There are several hypotheses about the pathogenesis of endometriosis. Retrograde menstruation involves the direct-contact deposition of endometrial tissue into the peritoneal cavity [8]. Retrograde menstruation leads to endometrial tissue seeding directly onto structures nearby the uterus. Coelomic metaplasia is another theory based on the embryonic origin of mesodermal organs such as the intestinal wall and female reproductive organs. Chronic inflammation and irritation of abdominopelvic structures can transform into endometrial tissue of similar embryonic origin. The embryonic rest theory hypothesizes that remnants of mullerian tissue remain in adult organs, and a change in the environment can proliferate cells that resemble fetal tissues [7,8].

Appendiceal endometriosis is present in 0.4% of the general population and 2.8% of patients with previously diagnosed endometriosis [9]. Endometrial tissue in the appendix is reported 66% of the cases in the serosa [9]. The endometrial tissue identified in this case reports exclusive involvement in the muscularis propria of both the appendix and the cecum without serosal deposits. This abnormal growth can progress to acute appendicitis with chronic inflammation caused by the endometrial glands and stroma, causing the development of fibrosis and hypertrophy of the muscularis propria and can wholly or partially occlude the appendiceal lumen [4]. It is unclear how this patient without any previous diagnosis of endometriosis developed endometriosis of the muscularis propria and puts into question the theory of retrograde menstruation as there can be no direct seeding without the involvement of the serosa. The appendix and the cecum are both mesodermal tissue, and the transformation of the intestinal wall supports the theory of coelomic metaplasia.

Presentation, diagnosis, and management of this disease are the same as appendicitis of any etiology. Patients will most commonly present with periumbilical pain migrating to the right lower quadrant with associated fevers, nausea, and diarrhea. The diagnosis is clinical; however, adjuncts such as computed tomography, magnetic resonance imaging, and ultrasonography are helpful in appropriate patient populations. The treatment is an appendectomy, and to confirm the diagnosis of appendiceal endometriosis, histopathological analysis of specimen is performed [10,11].

## Conclusions

Endometriosis of the appendix can present with right lower quadrant abdominal pain, which mandates an appendectomy. However, it is unusual to see evidence of endometrial tissue with the involvement of only the muscularis propria of the bowel wall without any previous history of endometriosis involving the abdominal cavity or any findings on the serosal layer. Appendiceal endometriosis is a histopathological diagnosis, and the surgeon should avoid a right hemicolectomy if confused with appendiceal cancer.
